# Use of Asynchronous Virtual Mental Health Resources for COVID-19 Pandemic–Related Stress Among the General Population in Canada: Cross-Sectional Survey Study

**DOI:** 10.2196/24868

**Published:** 2020-12-30

**Authors:** Chris G Richardson, Allie Slemon, Anne Gadermann, Corey McAuliffe, Kimberly Thomson, Zachary Daly, Travis Salway, Leanne M Currie, Anita David, Emily Jenkins

**Affiliations:** 1 Centre for Health Evaluation and Outcome Sciences Providence Healthcare Research Institute St Paul's Hospital Vancouver, BC Canada; 2 School of Nursing University of British Columbia Vancouver, BC Canada; 3 School of Population and Public Health University of British Columbia Vancouver, BC Canada; 4 Simon Fraser University Burnaby, BC Canada; 5 Patient Voices Network Vancouver, BC Canada

**Keywords:** virtual health, digital health, virtual mental health, mental health, public health, COVID-19, coping, stress, implementation, utilization

## Abstract

**Background:**

The COVID-19 pandemic has resulted in profound mental health impacts among the general population worldwide. As many in-person mental health support services have been suspended or transitioned online to facilitate physical distancing, there have been numerous calls for the rapid expansion of asynchronous virtual mental health (AVMH) resources. These AVMH resources have great potential to provide support for people coping with negative mental health impacts associated with the pandemic; however, literature examining use prior to COVID-19 illustrates that the uptake of these resources is consistently low.

**Objective:**

The aim of this paper is to examine the use of AVMH resources in Canada during the COVID-19 pandemic among the general population and among a participant subgroup classified as experiencing an adverse mental health impact related to the pandemic.

**Methods:**

Data from this study were drawn from the first wave of a large multiwave cross-sectional monitoring survey, distributed from May 14 to 29, 2020. Participants (N=3000) were adults living in Canada. Descriptive statistics were used to characterize the sample, and bivariate cross-tabulations were used to examine the relationships between the use of AVMH resources and self-reported indicators of mental health that included a range of emotional and coping-related responses to the pandemic. Univariate and fully adjusted multivariate logistic regression models were used to examine associations between sociodemographic and health-related characteristics and use of AVMH resources in the subgroup of participants who reported experiencing one or more adverse mental health impacts identified in the set of self-reported mental health indicators.

**Results:**

Among the total sample, 2.0% (n=59) of participants reported accessing AVMH resources in the prior 2 weeks to cope with stress related to the COVID-19 pandemic, with the highest rates of use among individuals who reported self-harm (n=5, 10.4%) and those who reported coping “not well” with COVID-19–related stress (n=22, 5.5%). Within the subgroup of 1954 participants (65.1% of the total sample) who reported an adverse mental health impact related to COVID-19, 54 (2.8%) reported use of AVMH resources. Individuals were more likely to have used AVMH resources if they had reported receiving in-person mental health supports, were connecting virtually with a mental health worker or counselor, or belonged to a visible minority group.

**Conclusions:**

Despite substantial government investment into AVMH resources, uptake is low among both the general population and individuals who may benefit from the use of these resources as a means of coping with the adverse mental health impacts of the COVID-19 pandemic. Further research is needed to improve our understanding of the barriers to use.

## Introduction

With growing global access to the internet [1], the World Health Organization [2] has identified digital health as a key health promotion strategy that can “benefit people in a way that is ethical, safe, secure, reliable, equitable and sustainable." Broadly, digital mental health comprises health care activities that are not carried out in traditional face-to-face format and may include synchronous (ie, in real time) video visits with mental health care providers or asynchronous (ie, not in real time) use of online self-management skill-building programs [3]. As a core aspect of the growth in digital health, there has been a recent surge in free, publicly available virtual mental health resources, including online programs and smartphone apps. In this paper, we use the term *asynchronous virtual mental health* (AVMH) *resources* to refer to any app, website, online tool, or other online support that does *not* involve synchronous direct contact with a mental health care provider. Although many of these publicly available mental health apps and online programs have been endorsed by users with generally high ratings and positive review comments [4], most are lacking direct scientific evidence of their effectiveness [5]. While not a replacement for professional decision making, AVMH resources nonetheless offer an alternative to in-person engagement with a mental health clinician, and aim to support people coping with symptoms related to a wide range of mental health conditions and challenges, including mild to moderate depression and anxiety, trauma, substance use, and other psychosocial stressors [6,7]. While limited, the research that has been conducted on smartphone and internet-based resources has demonstrated that AVMH resources can be effective in supporting people to manage their symptoms [8,9] and may reduce the need for clinical care [6].

The COVID-19 pandemic presents a heightened opportunity for AVMH resources to support mental well-being. The mental health impacts of COVID-19 are profound, including significant increases in depression, anxiety, and stress among the general population [10-13]. Public health measures, such as lockdown and quarantine, are necessary for reducing the spread of the virus but are also associated with negative psychological effects [14,15]. Loss of employment, anxiety about physical health and COVID-19 exposure, and social isolation are additional stressors that can contribute to poor mental health during the pandemic and beyond [16-18]. Further, researchers have warned of a likely increase in self-harm and suicide related to COVID-19 stressors [19,20]. In May 2020, many in-person mental health supports were reduced, suspended, or transitioned to online services in Canada, amid numerous reports from researchers around the globe calling for the rapid expansion of AVMH resources to address the mental health impact of the pandemic [21-24]. In the context of limited in-person services, AVMH resources represent a scalable means of promoting coping among those experiencing mental health challenges, including members of the general population who have not engaged with the mental health system but would likely benefit from these resources as a means of managing the mental health impacts of the COVID-19 pandemic [8,9,23].

Despite the potential for AVMH resources to support coping with COVID-19–related stressors, research suggests that uptake has been consistently low. While research examining AVMH resources in the context of COVID-19 is still emerging and no Canadian data are currently available, Yao and colleagues [25] reported results from a survey in China indicating that only 3.7% of respondents experiencing high levels of acute stress had used mental health services in any form in the early months of the COVID-19 outbreak. To our knowledge, few, if any, studies have examined the uptake of AVMH resources in the COVID-19 context. Prior to COVID-19, AVMH uptake and retention have been a perpetual challenge, with many internet-based resources and apps having very low use among the general population [26,27]. Moreover, use of AVMH resources tends to decline sharply in the first week following initial use; for example, Baumel and colleagues [28] found that as few as 4% of users of mental health apps continued to access the resource 15 days after initial installation.

In Canada, various AVMH programs have been promoted to support individuals’ mental health during the COVID-19 pandemic, including BounceBack [29], Wellness Together Canada [30], WellCan [31], and MindBeacon [32]. Free and available to the public, these resources hold promise for improving mental health and well-being at a time when in-person services are reduced and travel for services is limited, particularly for individuals in rural and remote areas. While a digital divide persists, particularly among low-income and rural populations [33], internet access is widespread in Canada, supporting the uptake of AVMH resources. In 2018, 94% of Canadians reported home internet access, and 88% of internet users had a smartphone [34]. AVMH resources have great potential to promote mental health and coping with stressors during the COVID-19 pandemic, given their demonstrated efficacy in supporting individuals’ self-management of a variety of mental health challenges [8,35]; however, little is known about the uptake of these resources among the general public in Canada in the pandemic context. Further, as mentioned, the global evidence on the use of similar virtual resources remains extremely limited. The aim of this investigation is to examine the use of AVMH resources during the COVID-19 pandemic among the general population in Canada to inform intervention research, development, and refinement of asynchronous virtual public health solutions related to mental health service delivery.

## Methods

### Overview

Data for this analysis were drawn from the first wave of a large multiwave cross-sectional monitoring survey, *Assessing the Impacts of COVID-19 on Mental Health*. This study utilizes a nationally representative sample of people aged ≥18 years living in Canada with the aim to examine mental health, stress, and coping during the COVID-19 pandemic. This project involves a unique partnership between academic researchers and 2 not-for-profit mental health organizations, the Canadian Mental Health Association (CMHA) and the Mental Health Foundation in the United Kingdom. Survey items were largely drawn from a repeated cross-sectional survey first commissioned by the Mental Health Foundation for COVID-19. Adaptations were made for the Canadian context.

### Data Collection

Wave 1 of the survey was distributed from May 14 to 29, 2020, by Maru/Matchbox, a national polling vendor that maintains the online Maru Voice Panel of 125,000 Canadian adults. Members of the panel were randomly invited to participate in the survey from national census–informed stratifications defined by sociodemographic characteristics (age, gender, household income, and region), with adjustments for response propensity. Maru/Matchbox utilized target sampling to ensure inclusion of populations that may be difficult to reach via the internet, including older adults and racialized populations. In total, 3558 participants were invited by email to complete the survey to yield a total of 3000 respondents (84% invitation-to-response rate). The survey was available in English and French, Canada’s two official languages. The data collection time period was chosen to correspond with the beginning phases of the reopening of many of Canada’s provinces and territories, following approximately 2 months of public health orders that resulted in closures and physical distancing after COVID-19 was declared a pandemic by the World Health Organization on March 11, 2020 [36]. Additional details on the survey development and implementation have recently been published [13].

### Measures and Analysis

Participants reported on their sociodemographic information as well as their mental health during the COVID-19 pandemic. Participants were asked to rate their mental health *now* compared to *before* the COVID-19 pandemic and related restrictions. Participants also reported on their overall coping and emotional responses to the COVID-19 pandemic in the past 2 weeks, including feeling depressed, stressed, hopeless, and anxious. Finally, participants reported if they had experienced suicidal thoughts/feelings or deliberate self-harm as a result of the COVID-19 pandemic in the past 2 weeks. These indicators were informed by research on previous pandemics with further refinement carried out via a citizen’s jury participatory methodology process involving people with lived experience of mental health conditions [37].

To assess the use of AVMH resources during COVID-19, participants responded to the survey item, “Which of the following have helped you cope with stress related to the COVID-19 pandemic in the past 2 weeks?” Participants were asked to select all that apply from a checklist of items that included three mental health strategies: (1) accessing virtual mental health resources (eg, online cognitive behavioral therapy, etc); (2) connecting with a mental health worker or counselor virtually (eg, via phone, video chat, etc); and (3) receiving in-person mental health supports. Study participants who endorsed the first option were classified as having used AVMH resources for this investigation.

Descriptive statistics were used to characterize the sample, and bivariate cross-tabulations were used to examine the relationships between self-reported indicators of mental health and the use of AVMH resources. Any participant who reported experiencing a negative impact of the pandemic on one or more of the self-reported mental health indictors was classified as experiencing an adverse mental health impact. The subgroup of participants who reported experiencing an adverse mental health impact included participants who reported that their mental health was now “slightly worse” or “significantly worse” compared to before the COVID-19 pandemic; participants who indicated that their level of coping with the stress of the COVID-19 pandemic was “not very well” or “not well at all”; and participants who reported feeling panicked, anxious or worried, hopeless, stressed, or depressed as a result of the COVID-19 pandemic. Additionally, respondents who reported* *experiencing suicidal thoughts/feelings as a result of the COVID-19 pandemic in the past 2 weeks and those who reported* *deliberately hurting themselves as a result of the COVID-19 pandemic in the past 2 weeks were also classified as experiencing an adverse mental health impact. Univariate and fully adjusted multivariate logistic regression models were then used to examine the associations between a set of a priori specified sociodemographic and health-related characteristics and use of AVMH resources in the subgroup of participants classified as experiencing an adverse mental health impact.

### Ethics

Ethical approval for this study was provided by the Behavioural Research Ethics Board at the University of British Columbia (H20-01273). Participants provided consent online prior to beginning the survey. Maru/Matchbox provided all participants with a small honorarium for completing the survey.

## Results

The sample comprised 3000 participants; detailed sociodemographic characteristics are presented in Table 1.

**Table 1:** Sociodemographic characteristics of the sample.

Characteristic		Total respondents (N=3000), n (%)^a^
**Gender^b^**		
	Male	1467 (48.9)
	Female	1533 (51.1)
**Age group (years)**		
	Youth (18-24)	62 (2.1)
	Adult (25-64)	2145 (71.5)
	Senior (≥65)	793 (26.4)
**Household income ($CAD)**		
	<$25,000	234 (7.8)
	$25,000-$50,000	504 (16.8)
	$50,000-$100,000	992 (33.1)
	>$100,000	1270 (42.3)
**Education**		
	High school or less	431 (14.4)
	Some university or college	1139 (38.0)
	University or college degree (diploma, undergraduate, and/or graduate degree)	1430 (47.7)
**Employment (multiple responses permitted)**		
	Working full time (≥30 hours per week)	1225 (40.8)
	Working part time (<30 hours per week)	286 (9.5)
	Retired	882 (29.4)
	Full-time student (eg, school, college, university, job training)	50 (1.7)
	Part-time student (eg, school, college, university, job training)	16 (0.5)
	Unemployed (due to COVID-19)	284 (9.5)
	Unemployed (prior to COVID-19)	103 (3.4)
**Ethnic minority^c^**		
	Not a visible minority	2128 (70.9)
	Visible minority	389 (13.0)
	Indigenous (eg, First Nations, Inuit, Métis)	87 (2.9)
**Province**		
	British Columbia/territories	440 (14.7)
	Alberta	333 (11.1)
	Ontario	1140 (38.0)
	Quebec	658 (21.9)
	Manitoba/Saskatchewan	194 (6.5)
	Atlantic provinces	235 (7.8)
**Location**		
	Urban	2516 (83.9)
	Rural	484 (16.1)
Self-reported prior mental health condition (yes)		546 (18.2)
Accessed virtual mental health resources in the past 2 weeks (yes)		59 (2.0)

^a^Note that some category percentages do not add up to 100 due to missing responses.

^b^Maru/Matchbox, the polling vendor that distributed this survey, provides demographic data for each panel member collected prior to survey completion. Though this binary variable was used in this analysis, the research team recognizes that binary gender identities do not accurately reflect many individuals’ self-identified gender.

^c^Visible minority and non–visible minority categories were constructed by the research team based on participants’ self-reported ethnicity (eg, South Asian, Middle Eastern, European origins).

Among the total sample, 59 (2.0%) participants reported accessing AVMH resources in the prior 2 weeks to cope with stress related to the COVID-19 pandemic. Table 2 presents rates of self-reported AVMH resource use or nonuse across various self-reported mental health indicators. The highest rate of access was among individuals who reported having deliberately hurt themselves (n=5, 10.4%), those who reported coping “not well” with stress related to the COVID-19 pandemic (n=22, 5.5%), those who experienced suicidal thoughts/feelings (n=14, 8.0%), and those who reported having “worse” mental health compared to before the COVID-19 pandemic (n=40, 3.6%). There was also significant overlap across these subgroups in that 74.3% (n=130) of participants who reported experiencing suicidal thoughts/feelings and 54.2% (n=26) of participants who reported deliberately hurting themselves also reported having worse mental health now compared to before the pandemic. Though higher than the sample average, accessing AVMH resources remained low among those who experienced negative emotions related to COVID-19, including individuals who felt hopeless (n=16, 4.5%), depressed (n=29, 4.3%), panicked (n=10, 4.3%), stressed (n=32, 2.9%), and anxious (n=38, 2.8%).

To identify sociodemographic and health-related characteristics associated with reported use of AVMH resources, a subgroup was created that contained any participant who reported experiencing one or more adverse mental health impacts on the mental health indicators listed in Table 2. This subgroup comprised 1954 participants, of which 54 (2.8%) reported use of AVMH resources. Table 3 presents the results of a multivariate logistic regression model examining the association between sociodemographic characteristics and the use of AVMH resources within this subsample of participants who reported adverse mental health outcomes due to the COVID-19 pandemic.

Results of the fully adjusted multivariate logistic regression model indicate that, among the subsample who reported adverse mental health impacts due to COVID-19, those who reported receiving in-person mental health supports or connecting virtually with a mental health worker or counselor were significantly more likely to report accessing AVMH resources (odds ratio [OR] 6.05, 95% CI 1.35-27.17 and OR 8.96, 95% CI 4.36-18.42, respectively). Additionally, individuals belonging to visible minority groups were more likely to report accessing AVMH resources (OR 3.79, 95% CI 1.83-7.86).

**Table 2:** Accessing asynchronous virtual mental health (AVMH) resources across indicators of self-reported mental health impact.

Mental health indicator		Used AVMH resource, n (%)	Did not use AVMH resource, n (%)
**Compared to before the COVID-19 pandemic and related restrictions in Canada, how would you say your mental health is now?^a^**			
	About the same or better (n=1874)	19 (1.0)	1855 (99.0)
	Worse (n=1121)	40 (3.6)	1081 (96.4)
**Overall, how well do you think you are coping with stress related to the COVID-19 pandemic?^a^**			
	Fairly or very well (n=2439)	36 (1.5)	2403 (98.5)
	Not well (n=400)	22 (5.5)	378 (94.5)
**Felt panicked as a result of the COVID-19 pandemic in the past 2 weeks^b^**			
	Yes (n=231)	10 (4.3)	221 (95.7)
	No (n=2769)	49 (1.8)	2720 (98.2)
**Felt anxious or worried as a result of the COVID-19 pandemic in the past 2 weeks^a^**			
	Yes (n=1361)	38 (2.8)	1323 (97.2)
	No (n=1639)	21 (1.3)	1618 (98.7)
**Felt hopeless as a result of the COVID-19 pandemic in the past 2 weeks^a^**			
	Yes (n=353)	16 (4.5)	337 (95.5)
	No (n=2647)	43 (1.6)	2604 (98.4)
**Felt stressed as a result of the COVID-19 pandemic in the past 2 weeks^a^**			
	Yes (n=1103)	32 (2.9)	1071 (97.1)
	No (n=1897)	27 (1.4)	1870 (98.6)
**Felt depressed as a result of the COVID-19 pandemic in the past 2 weeks^a^**			
	Yes (n=676)	29 (4.3)	647 (95.7)
	No (n=2324)	30 (1.3)	2294 (98.7)
**Experienced suicidal thoughts/feelings in the past 2 weeks^a^**			
	Yes (n=176)	14 (8)	162 (92)
	No (n=2792)	43 (1.5)	2749 (98.5)
**Deliberately hurt myself in the past 2 weeks^a^**			
	Yes (n=48)	5 (10.4)	43 (89.6)
	No (n=2936)	52 (1.8)	2884 (98.2)

^a^*P*<.01, based on chi-square analyses or exact test (Fischer or Fisher-Freeman-Halton).

^b^*P*<.05, based on chi-square analyses or exact test (Fischer or Fisher-Freeman-Halton).

**Table 3:** Results of univariate and fully adjusted multivariate logistic regression models examining the association between use of asynchronous virtual mental health resources and sociodemographic characteristics among participants who reported adverse mental health impacts related to the COVID-19 pandemic (n=1954).

Variable		Unadjusted odds ratio (95% CI)	Adjusted odds ratio (95% CI)
**Age group (reference: youth)**			
	Adult	0.70 (0.16-2.96)	1.13 (0.24-5.44)
	Senior	0.27 (0.05-1.41)	0.31 (0.04-2.67)
Gender (female)		1.23 (0.71-2.14)	1.38 (0.70-2.74)
LGBTQ2+^a^ (yes)		2.09 (0.97-4.52)	1.42 (0.58-3.50)
**Income (reference: >$100,000)**			
	<$25,000	1.49 (0.59-3.78)	0.63 (0.17-2.32)
	$25,000-$50,000	1.33 (0.61-2.87)	0.84 (0.31-2.25)
	$50,000-$100,000	1.16 (0.61-2.21)	1.01 (0.48-2.10)
**Visible minority (reference: no)**			
	Yes	3.59 (1.95-6.61)	3.79 (1.83-7.86)^b^
	Indigenous	1.79 (0.42-7.73)	1.69 (0.35-8.28)
Location (rural)		0.69 (0.29-1.63)	1.08 (0.39-3.04)
**Highest level of education completed (reference: university)**			
	High school or less	0.41 (0.12-1.36)	0.52 (0.14-1.98)
	Some university or college	1.07 (0.61-1.87)	1.01 (0.50-2.03)
Disability (yes)		1.75 (0.86-3.52)	1.38 (0.54-3.53)
Pre-existing mental health condition (yes)		2.97 (1.72-5.12)^b^	1.69 (0.80-3.58)
Receiving in-person mental health supports (yes)		11.61 (3.66-36.87)^b^	6.05 (1.35-27.17)^c^
Connecting with a mental health worker or counselor virtually (eg, via phone, video chat, etc) (yes)		12.19 (6.68-22.24)^b^	8.96 (4.36-18.42)^b^

^a^LGBTQ2+: lesbian, gay, bisexual, transgender, queer/questioning, and two-spirited.

^b^*P*<.01.

^c^*P*<.05.

## Discussion

### Principal Findings

Findings from this study illustrate that the reported uptake of AVMH resources was extremely low (2.0%) among the general population in Canada in the context of the first 3 months of the COVID-19 pandemic. A similarly low level of use (2.8%) was observed in the subgroup of participants who reported an adverse mental health impact related to COVID-19. While utilization of these resources was higher among particular groups experiencing mental health challenges as a result of the pandemic, including those who had engaged in self-harm behaviors, experienced suicidal thoughts, or reported poor coping, uptake remained low even among these groups. While representing a different national context for mental health care, these findings are consistent with Yao and colleagues’ [25] report of very low use of any mental health services among adults in China experiencing high levels of acute stress during COVID-19. Despite evidence indicating efficacy of select resources [38,39], promotion of existing AVMH resources (eg, [40]), and numerous calls for the expansion of these resources among researchers and public health officials, there is a dearth of evidence regarding the uptake of AVMH resources among the general public and population subgroups experiencing adverse mental health impacts during the current pandemic. This study is among the first to present data regarding the extent to which members of the general public report accessing AVMH resources during the COVID-19 pandemic, while also examining access among a subgroup of individuals experiencing adverse mental health impacts related to the pandemic. In this section, we discuss key findings from this study: the low uptake of AVMH resources within the sample, higher use among the visible ethnic minority subgroup and those who accessed other forms of mental health support, and limited use of AVMH resources among those who reported self-harm or suicidal thoughts.

COVID-19 has presented unexpected and significant stressors that have been demonstrated to negatively impact mental health worldwide [10,12]. While for some individuals, the mental health impacts are considerable, including posttraumatic stress disorder, self-harm, and suicidal ideation [20,41], most people will experience mild to moderate symptoms of stress, anxiety, and low mood [11,42,43]. Such subclinical symptoms have been shown to be particularly responsive to self-directed mental health interventions, including virtual resources [44]. Research also indicates that accessing internet-based cognitive behavioral therapy for depression is associated with reductions in suicidal ideation [45], suggesting that use of AVMH resources can also contribute to reductions in these adverse mental health experiences. In the context of COVID-19 public health measures, including lockdown and physical distancing, mental health apps and online programs are particularly important public resources that are free or low cost, widely available, and can support mental health without additional risks for spreading the virus. However, research demonstrates that prior to COVID-19, uptake of AVMH resources was consistently low [26,28]. Our findings illustrate that despite the increased value of virtual supports during the pandemic and promotion of AVMH resources in Canada, use of these resources remains extremely limited within the general population. Concerningly, use remains low (<10.5%) among population subgroups who would appear to benefit from their use, including those experiencing suicidal thoughts, anxiety, hopelessness, stress, and depression related to COVID-19. While many companies and organizations are producing AVMH resources, there is an expectation of “build it and they will come,” but that does not appear to be the case.

Previous literature on the challenges of AVMH resources sheds light on potential reasons for poor uptake in the context of COVID-19. Many users have reported technological issues that hinder the usability of the apps, including poor speed or stability of their internet connection [46]. Additionally, potential users may be deterred by privacy and confidentiality concerns [6,47] or by a lack of comfort and familiarity with the internet and smartphone devices, such as among older adults [48]. While the AVMH resources promoted by governments and public health officials in Canada to support mental health during COVID-19 are evidence based and reputable, many apps advertised online or through app stores are not grounded in evidence or validated through research and may not actually be effective for managing mental health challenges [6,22]. Further research is needed to examine the barriers to widespread uptake of AVMH resources, particularly among individuals experiencing mental health impacts due to the COVID-19 pandemic. Such research can guide refinement of existing AVMH resources and promotion strategies for these resources among the general public and those experiencing mental health challenges.

In addition to presenting findings on accessing of virtual mental health supports among the general population, findings from this study illustrate that a higher proportion of specific population subgroups reported using these resources. We found that belonging to a visible minority ethnic group was associated with an increased likelihood of accessing AVMH resources, even after adjusting for age, income, and education. A potential factor in increased use among these populations may be heightened stigma toward mental health challenges within certain cultures. For example, cultural values held by some individuals of Asian descent have contributed to higher stigma about mental health challenges and reduced help seeking for in-person mental health supports [49,50]. Research has further suggested that while individuals of Asian descent may exhibit fewer mental health help-seeking behaviors overall, there is evidence of an increased preference among Asian American college students (compared to Caucasian students) for online-only supports versus face-to-face services [51]. It is possible that the anonymity of virtual mental health supports may therefore facilitate increased use among particular visible minority populations, as a preferred alternative to seeking one-on-one or group support from a mental health clinician.

Additionally, individuals who were either receiving in-person mental health supports or connecting virtually with a mental health worker or counselor were significantly more likely to report having accessed AVMH supports. This suggests that those who seek support may be motivated to try multiple strategies for supporting their own mental health, or that mental health clinicians may be directing clients toward AVMH resources to augment individual therapy. Previous literature demonstrates that integrating the use of virtual resources with other forms of mental health care, such as discussing trends from a mental health app with a clinician during an in-person appointment, can lead to improved outcomes [52]. As such, enhancing clinicians’ awareness of AVMH resources and providing training specifically for clinicians on how to integrate these resources into mental health care, may increase uptake among individuals who may benefit from the support they provide. Research has further demonstrated that while most users do not sustain engagement after initial sign-up [27], supports built into the system to facilitate engagement can be effective. Specifically, uptake and retention are significantly higher among users of AVMH resources who receive peer support [27], and automated conversational agents such as chatbots have been shown to effectively support sustained engagement [53]. While clinician and peer support may be logistically challenging and costly, Burger and colleagues [54] note that current AVMH resources “do not get close to the full technological potential of e-mental health” and that enhancing the available features of these resources may lead to greater uptake and retention of users.

While AVMH resources were underutilized across the entire sample, including among those experiencing mental health challenges, what was particularly concerning was the relatively low uptake among those who had deliberately self-harmed or had suicidal thoughts/feelings in the past 2 weeks. While specific AVMH resources related to suicide are fewer compared to those addressing mental health challenges such as low mood and anxiety [55], many resources address a broad range of mental health concerns that may underlie self-harm or suicidal ideation. Although a recent systematic review and meta-analysis concluded that internet-based interventions directly targeting suicidal ideation using cognitive behavioral therapy represent a promising means of providing low-threshold support to address suicidal ideation [56], researchers have suggested that AVMH resources may not represent the most appropriate form of mental health support for individuals with this experience given the severity of symptoms and degree of risk involved [57]. Despite relatively low uptake, use of mental health resources among individuals who reported self-harm or suicidal ideation was significantly higher compared to the general population, suggesting a desire for support seeking in this population. As Frost and Casey [58] suggest, utilizing virtual resources may be a critical stepping stone toward engaging in more intensive in-person clinical support.

### Limitations

The sampling approach used in this study aimed to generate a large nationally representative sample of individuals living in Canada. The representativeness of the sample was supported since, according to the results from the 2016 Canadian census [59], 77.7% of the population did not belong to a visible minority (versus 70.9% in our sample), and 18.7% of the population was considered rural (versus 16.1% in our sample). In terms of household income, 57.7% of individuals reported an annual household income that was less than $100,000, which is consistent with recent data from Statistics Canada indicating that the average household income in Canada in 2017 was $93,300 [60]. However, while the sample was representative in these ways, there are other ways in which it did not reflect the overall population of Canada. For example, because it was an online survey, only individuals with internet access were able to participate. Individuals who were unable to afford the cost of internet service or equipment, or those living in rural “blackout zones” are therefore not represented in our sample [34,61]. As such, the actual use of AVMH resources among the general population may be even lower than that reflected in our findings.

The indicators of mental health used in the survey did not include clinical assessments of mental illness nor did it include prepandemic measures of baseline mental health. However, as noted in the description of study methods, the selection of items was informed by research on previous pandemics and the items were refined in consultation with people with lived experience of mental health conditions through a citizen’s jury participatory methodology process [37]. Additionally, accessing AVMH resources was assessed in this survey through a single item measure. We are therefore unable to present data on which AVMH resources were used (eg, websites, apps, podcasts), participants’ perceptions of the effectiveness of the resources, length of use, or why they may have stopped using these resources. Additionally, our survey asked participants about accessing AVMH resources to cope with COVID-19 stress and may not have captured use of these resources for other reasons, such as to help manage other mental health challenges. To further examine the use of AVMH resources among people living in Canada, we have expanded our survey content for subsequent waves of data collection to add further depth and nuance to the results presented in this investigation. It is also important to note that the cross-sectional design of this study prevents us from making causal conclusions. However, we will be conducting future waves of data collection over the course of the pandemic that will help address this limitation.

### Comparison With Prior Work

While multiple researchers have highlighted the importance of virtual resources to address the significant mental health impacts of the COVID-19 pandemic, there remains a paucity of data on their use, uptake, and retention. However, previous research suggests that these resources may be underutilized [28], and emerging data within the COVID-19 context suggest low uptake among acutely stressed members of the general population [25]. Our examination of the use of AVMH resources related to COVID-19 stressors among people living in Canada is one of the first to provide empirical evidence on trends in accessing AVMH resources among the general population, with particular attention to their uptake among people experiencing adverse mental health impacts related to the current COVID-19 pandemic. Our analysis illustrates that not only is overall use of AVMH resources low, but use remains low even among individuals who would be predicted to benefit from these resources, such as individuals experiencing negative emotions (eg, anxiety) related to COVID-19 and individuals experiencing worsening mental health and difficulty coping.

### Conclusions

AVMH resources hold great promise to support mental well-being during the COVID-19 pandemic by helping individuals cope with stressors and negative mental health impacts. However, despite efforts to promote AVMH resources within Canada, the results from this study suggest that many individuals who may benefit from these resources are not accessing them to help manage mental health challenges. Further research that examines the barriers and facilitators to AVMH resource use is needed to enhance uptake and impact.

## Abbreviations

AVMH: asynchronous virtual mental health
CMHA: Canadian Mental Health Association
OR: odds ratio
